# [Retracted] 3,4‑Dihydroxyphenylethanol alleviates early brain injury by modulating oxidative stress and Akt and nuclear factor‑κB pathways in a rat model of subarachnoid hemorrhage

**DOI:** 10.3892/etm.2024.12734

**Published:** 2024-09-30

**Authors:** Peng Fu, Quan Hu

Exp Ther Med 11:1999–2004, 2016; DOI: 10.3892/etm.2016.3101

Following the publication of this paper, it was drawn to the Editor’s attention by a concerned reader that certain of the western blot protein bands featured in Figs. 5 and 6 on p. 2002 were, in terms of their shape, strikingly similar to bands appearing elsewhere in these figures, and this phenomenon coincided with possible breaks in the continuity of the gels that could be observed in their backgrounds.

The authors were asked for an explanation to account for these concerns, but the Editorial Office did not receive a reply. Therefore, given the lack of responsiveness of the authors and owing to a lack of confidence in the presentation of the abovementioned data in this paper, the Editor of *Experimental and Therapeutic Medicine* has decided that it should be retracted from the Journal. The Editor apologizes to the readership for any inconvenience caused.

